# Highly preserved consensus gene modules in human papilloma virus 16 positive cervical cancer and head and neck cancers

**DOI:** 10.18632/oncotarget.23116

**Published:** 2017-12-07

**Authors:** Xianglan Zhang, In-Ho Cha, Ki-Yeol Kim

**Affiliations:** ^1^ Department of Pathology, Yanbian University Medical College, Yanji City, Jilin Province, China; ^2^ Oral Cancer Research Institute, College of Dentistry, Yonsei University, Seoul, Korea; ^3^ Department of Oral and Maxillofacial Surgery, College of Dentistry, Yonsei University, Seoul, Korea; ^4^ Dental Education Research Center, BK21 PLUS Project, College of Dentistry, Yonsei University, Seoul, Korea

**Keywords:** gene expression, consensus module, head and neck cancer, cervical cancer, multicancer therapy

## Abstract

In this study, we investigated the consensus gene modules in head and neck cancer (HNC) and cervical cancer (CC). We used a publicly available gene expression dataset, GSE6791, which included 42 HNC, 14 normal head and neck, 20 CC and 8 normal cervical tissue samples. To exclude bias because of different human papilloma virus (HPV) types, we analyzed HPV16-positive samples only. We identified 3824 genes common to HNC and CC samples. Among these, 977 genes showed high connectivity and were used to construct consensus modules. We demonstrated eight consensus gene modules for HNC and CC using the dissimilarity measure and average linkage hierarchical clustering methods. These consensus modules included genes with significant biological functions, including ATP binding and extracellular exosome. Eigengen network analysis revealed the consensus modules were highly preserved with high connectivity. These findings demonstrate that HPV16-positive head and neck and cervical cancers share highly preserved consensus gene modules with common potentially therapeutic targets.

## INTRODUCTION

Differential gene expression analysis has been widely used to identify critical gene and pathways involved in tumorigenesis [1–3]. However, a part from differential gene expression data, there is tremendous amount of critical information in the gene expression datasets that is ignored. For example, some mutant proteins with altered functions show similar expression in diseased and healthy individuals [3]. Therefore, diagnosis or prognosis based on the expression of a single biomarker gene may not be reliable. This implies that differential co-expression and differential network analysis are more relevant as they help in understanding the underlying biological processes that are key to the disease understanding and therapy [3–8].

Another strategy to optimize gene expression data involves comparative and integrative analyses of gene expression in multiple cancer types [9, 10]. The integrative approach improves reproducibility and identifies common markers for multiple types of cancer. The multi-cancer biomarkers are more reliable and superior than cancer-specific biomarkers [11]. Genes or proteins that are directly linked are most likely to belong to the same biological pathway or function [12]. Such groups of genes or proteins that belong to the same biological pathway are called modules. A common module that is found in multiple cancers is defined as the consensus module.

Squamous cell carcinoma (SCC) is the most common histological type of head and neck cancer (HNC) and cervical cancer (CC). Both HNC and CC demonstrate similar multistep progression, where in normal squamous epithelial cells undergo dysplastic changes followed by carcinoma formation, which subsequently becomes invasive and metastatic. Moreover, human papilloma virus type 16 (HPV16) is a major pathogen in HNC and CC [13]. Therefore, in this study, we investigated consensus gene modules of HNC and CC to identify common targets for therapy of HPV16-positive HNC and CC.

## RESULTS

### Common differentially expressed genes of HNC and CC

The GSE6791 microarray dataset was analyzed to identify common genes that play a significant role in HNC and CC. The dataset included 54675 probes and 84 samples (42 HNC and14 normal head and neck samples as well as 20 CC and 8 normal cervical tissue samples). We analyzed only HPV16 positive samples to exclude bias because of different HPV types.

We identified significantly expressed genes separately in HNC and CC, based on Mann-Whitney U test. The rates of commonly identified genes were 43.8% and 28.9% of the detected significant genes from HNC and CC, respectively. This indicated that these two types of cancers might have similar genomic variations to some extent.

**Figure 1:**
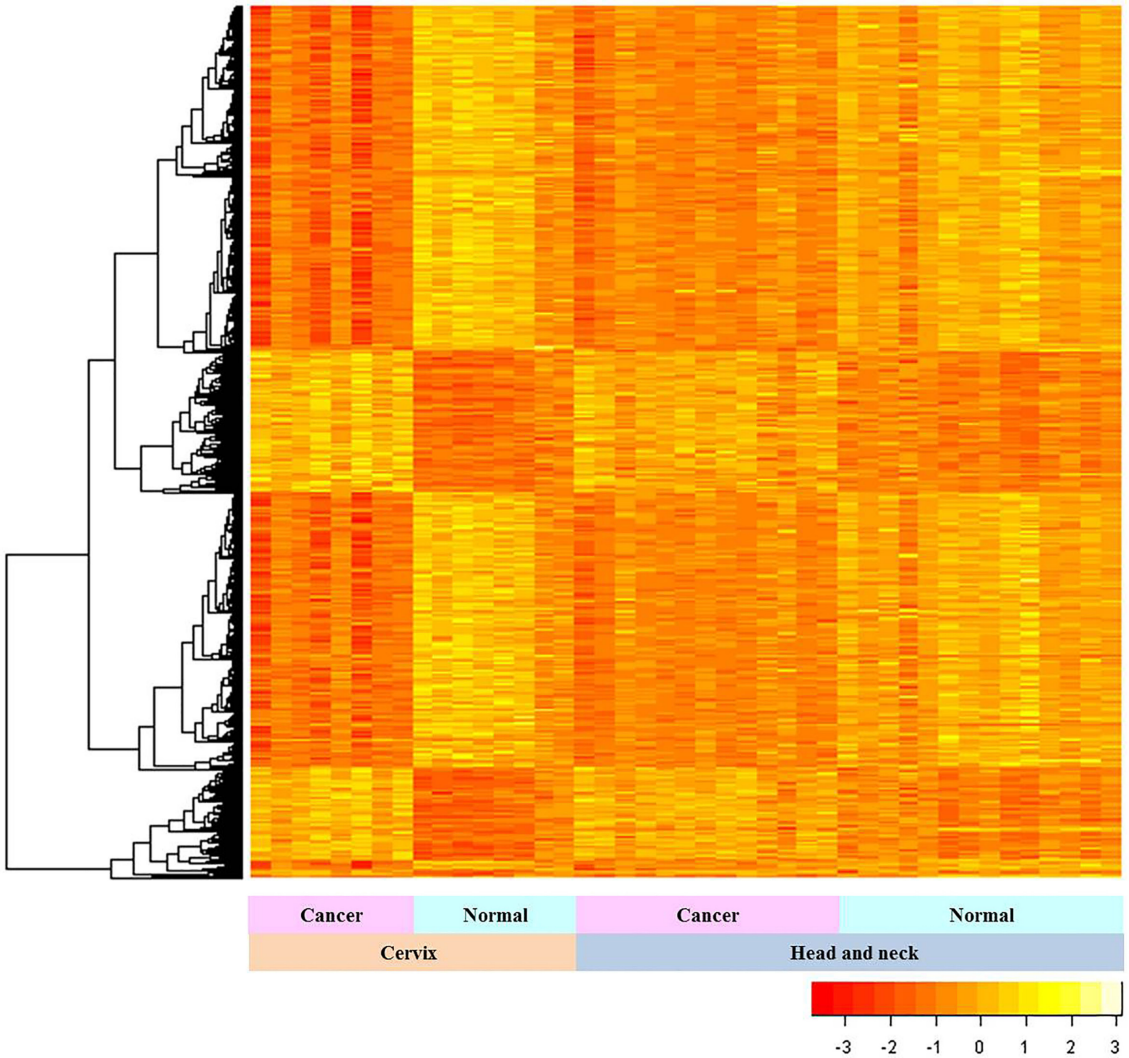
Gene expression patterns in HNC and CC The expression patterns of 3824 common genes in HNC and CC samples relative to their corresponding controls are shown. The statistical analysis was performed with Mann-Whitney U test. The vertical and horizontal axes represent the gene lists and samples, respectively.

Among 3824 complete genes, 977 genes with high connectivity were used to construct the consensus module. The expression patterns of common genes in HNC and CC samples were analyzed by unsupervised hierarchical clustering (Figure 1; orange indicates low and yellow indicates high expression). The genes were classified into 2 groups based on their expression patterns in HNC and CC samples compared to their respective normal samples (Figure 1). Genes that were downregulated in HNC and CC samples than normal samples were clustered into one group, whereas genes that were upregulated in the two cancer types than corresponding normal samples were clustered into another group (Figure 1).

### Consensus modules in HNC and CC

**Figure 2:**
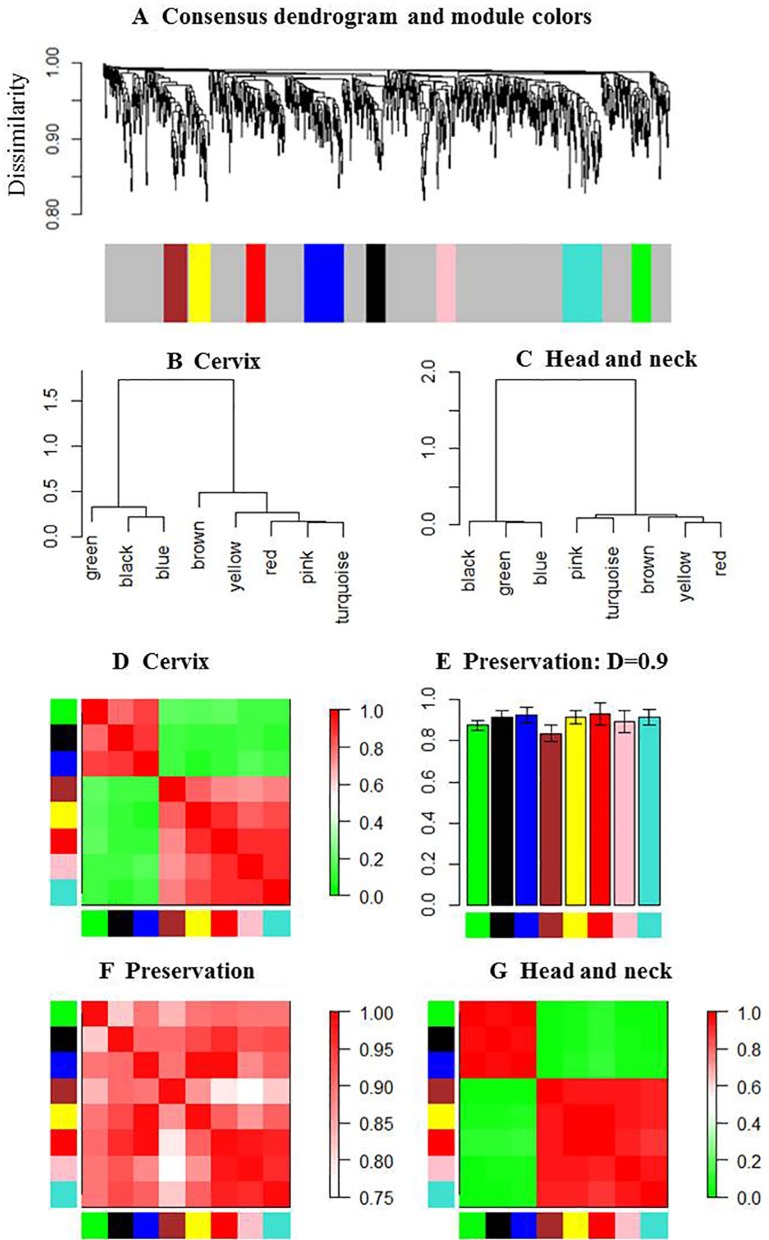
Eigengene network in HNC and CC (**A**) Dendrogram shows hierarchical clustering of genes used to identify the consensus modules in HNC and CC samples. Branches of the dendrogram correspond to consensus modules. Genes in each module are assigned a particular color code, which is shown below the dendrogram. Genes not assigned to any of the modules are colored grey. (**B** and **C**) Clustering dendrograms show consensus module eigengenes in HNC and CC. The two major modules are evident in both dendrograms. (**D**) Heatmap shows eigengene proximities in the consensus eigengene network for CC samples. Each row and column corresponds to one eigengene (labeled by consensus module color). In the heatmap, red denoteshigh proximity (positive correlation) and green denotes low proximity (negative correlation). (**E**) Bar graph showing preservation measure (*D*) for each consensus eigengene (vertical axes). The module color is represented in each bar for the corresponding eigengenes. (**F**) Heatmap shows proximities in the preservation of eigengene networks of HNC and CC modules. Each row and column corresponds to a consensus module. The red pattern reveals the proximity of specific module in HNC and CC. (**G**) Heatmap shows eigengene proximities in the consensus eigengene network for HNC samples. The 8 consensus modules were clearly merged into two modules in HNC.

Next, we used the dissimilarity measure and average linkage hierarchical clustering method to construct consensus modules with common genes between HNC and CC [7, 12]. Genes in similar consensus modules were assigned a color code, whereas unassigned genes were colored grey. As shown in Figure 2A, we identified eight consensus modules that were assigned specific color codes, namely, brown (83 genes), yellow (80 genes), blue (105 genes), turquoise (141 genes), green (62 genes), red (53 genes), black (45 genes), pink (40 genes) and grey (368 genes).

The modules were characterized by height and minimum size of branch. Consensus modules represent biological pathways shared between the HNC and CC data sets. For each data set, we represented the consensus modules by their corresponding eigengenes and then constructed a eigengene network (Figure 2). The consensus eigengenes in HNC and CC groups belonged to one of two branches (Figure 2B–2C). The green, black and blue modules formed the first branch, whereas the brown, yellow, red, pink and turquoise modules formed the second branch. The module eigengenes were highly preserved (Figure 2). The eigengene networks of HNC and CC are shown in Figure 2D and 2G, respectively. The high connectivity showed that each individual eigengene in a module was highly preserved relative to the other eigengenes. The preservation indices were 0.811, 0.938, 0.933, 0.92, 0.835, 0.963, 0.92 and 0.919 for the brown, yellow, blue, turquoise, green, red, black and pink modules, respectively, with the overall preservation of 0.90 (Figure 2E). The consensus modules were preserved between the two data sets (Figure 2F).

### Gene expression patterns of eight consensus modules

**Figure 3:**
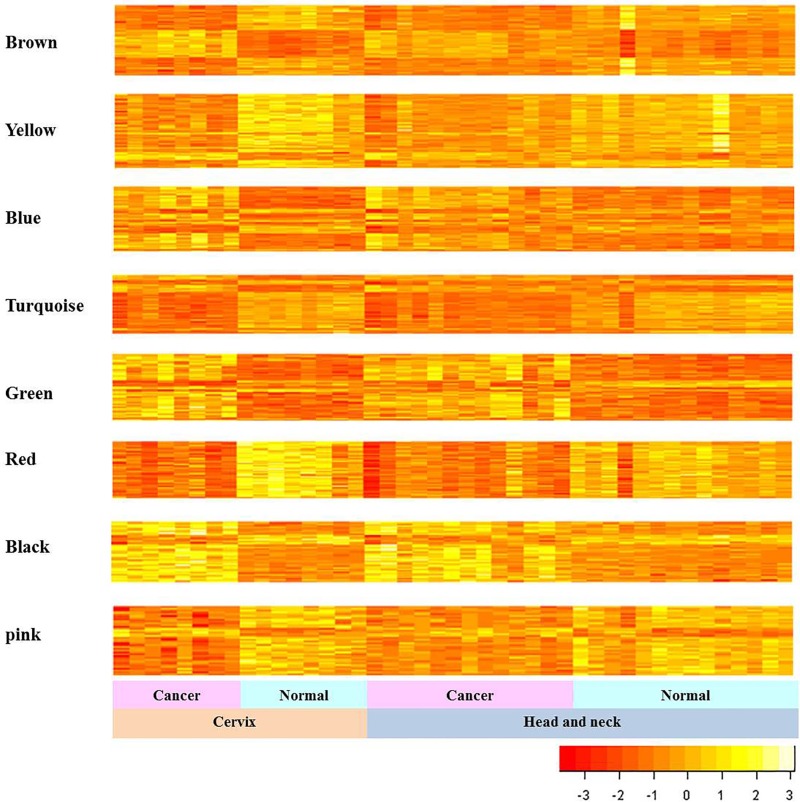
Gene expression patterns of consensus modules in HNC and CC The expression patterns of genes in the consensus modules of HNC and CC samples are shown. The vertical and horizontal axes of heat map represent gene expressions and samples, respectively.

Next, we explored the gene expression patterns of the 8 consensus gene modules between the cancer and their corresponding normal samples (Figure 3). We observed much clearer distinct differences in expression patterns between CC and normal cervical samples comparing to the differences between HNC and their corresponding normal head and neck samples. The genes in the 8 consensus modules are shown in Table 1 and Supplementary Table 1.

**Table 1:** Gene lists of eight consensus modules

Brown	Yellow	Blue	Turquoise	Green	Red	Black	Pink
LRRN4CL	TRIM40	CDC42SE2	KRT78	KLF7	TMEM72	TYMS	HYDIN2
NAV3	HOXC12	CNOT6L	LRRC43	STAT1	LOC285300	LOC375196	CCDC26
STMN1	MGC20647	FLJ31715	ATP6V1C2	CTSC	TP53BP1	DHFR	C18orf20
CACYBP	C21orf15	OSGIN2	C1orf177	CTSL1	F11	DHFR.2	OLFM3
DHFR	NFYC	EME2	C5orf28	SLC16A1	KDM5B	STIM1	C9orf68
SMAD2	LOC338694	RPAP3	CTNND1	CDKN2A	ACAA2	RNASEH2A	OR2C3
CDK1	CHML	LOC283888	RABEPK	CFB	TAOK2	RFC5	RNASET2
FAIM2	C6orf174	NEDD1	GBP6	IFI27	RAB3A	CDC45	C10orf44
MAPT	C9orf29	KIAA1543	HCG22	PLSCR1	SLC14A1	CLUAP1	LOC729870
ZWINT	KRT79	CBX3	LOC441178	PLOD2	INS	NFE2L3	RNF103
HSD11B2	SLC39A6	GNG10	TRIP10	LTBP1	IFNW1	MLF1	SAMD5
PDE2A	DGCR2	MED13	HOPX	LAMA3	PSG4	CXCL13	BHLHB9
PMAIP1	FAM20B	TNFAIP3	C9orf169	LOX	CLDN6	SYNGR3	DDX19A
ACOX2	SMAD2	IL8	TCP11L2	LDOC1	SELPLG	RIBC2	MLEC
BRCA1	SLC13A2	BCAS2	FMN1	MMP1	FAM120A	SYCP2	MLF1
NEK2	DAPK3	KIF2A	EMP1	HOMER3	PBX2	HOXC6	ZNF135
ABCA8	CA12	NFIL3	SOX4	HLF	PATZ1	ATP4A	ARMCX4
TGFBR3	ST3GAL2	NDST2	IFI30	PLAU	IGHA	CBX5	PDIA4
CKM	PRIM2	SLC43A1	FARP1	ISG15	POU2F2	NEDD4L	KCNK2
TTK	HSPB2	GAS1	MTMR3	MMP10	FSHB	OLFM1	RGSL1
PLK4	HOXD3	RECQL	LYN	PLA2G7	CYP11B2	APITD1	GABARAPL3
CLEC3B	ALOX15B	TMEFF1	ITPR2	PAK2	PVT1	HSP90AA1	C1orf216
PHYHIP	GCKR	SNX4	AKAP2	APOL1	KIR2DL1	IPO9	RAB7A
CCL14	FOXE1	CASK	C1QB	NCF2	KLF11	DTL	OR2J3

Twentyfour genes for each module were shown in the table and the order is insignificant.

### Annotation of gene ontology (GO) terms and KEGG pathways of eight consensus modules

Table 2 shows the annotation of the genes in the 8 consensus modules using GO terms and KEGG pathways with DAVID gene annotation tool (http://david.abcc.ncifcrf.gov/). We determined the *P*-values (modified Fisher exact *p*-value) and the Benjamini-Hochberg false discovery rate (FDR) to determine the significance of enrichment of the annotated terms. Red and black modules represent the key GO terms and KEGG pathways with significant Benjamini adjusted *P*-values (Table 2). These two modules were clearly distinct and showed high connectivity (red = 0.963, black = 0.920; Figure 2B–2C).

**Table 2:** KEGG pathways and GO terms of identified consensus modules for head and neck cancer and cervical cancer

Module	Enriched terms associated with gene list in module	Category	*P*-value^*^	Benjamini^**^
Brown	Fanconi anemia pathway Cell cycle	KEGG-pathway	1.6E-3 2.0E-2	1.1E-1 5.1E-1
	ATP bindin cytoplasm	GOTERM	4.4E-4 1.6E-3	3.6E-2 1.3E-1
Yellow	anterior/posterior pattern specification negative regulation of glucokinase activity regulation of neuronal synaptic plasticity	GOTERM	1.8E-3 1.8E-2 4.8E-2	4.4E-1 9.4E-1 9.9E-1
Blue	Nucleus endocytosis regulation of transcription, DNA-templated catalytic step 2 spliceosome	GOTERM	2.3E-3 4.0E-3 4.4E-3 4.6E-3	1.6E-1 6.4E-1 4.3E-1 1.6E-1
	Hedgehog signaling pathway	KEGG-pathway	9.8E-2	9.9E-1
Turquoise	Serotonergic synapse Insulin signaling pathway Oocyte meiosis Rap1 signaling pathway Long-term depression	KEGG-pathway	3.4E-3 7.3E-3 2.3E-2 3.0E-2 4.5E-2	4.1E-1 4.3E-1 7.0E-1 6.9E-1 7.6E-1
	extracellular exosome	GOTERM	8.5E-5	1.4E-2
Green	Herpes simplex infection Pathways in cancer	KEGG-pathway	2.2E-2 3.9E-2	8.0E-1 7.6E-1
	type I interferon signaling pathway defense response to virus	GOTERM	2.5E-5 7.7E-5	6.5E-5 1.5E-2
Red	Antigen processing and presentation Natural killer cell mediated cytotoxicity Cell adhesion molecules (CAMs)	KEGG-pathway	6.3E-11 2.9E-9 8.0E-2	3.3E-9 7.8E-8 7.7E-1
	regulation of immune response immune response	GOTERM	7.2E-4 2.2E-2	2.0E-1 9.7E-1
Black		GOTERM_	6.4E-6	1.2E-3
	regulation of transcription involved in G1/S transition of mitotic cell cycle G1/S transition of mitotic cell cycle DNA replication tetrahydrofolate metabolic process		5.7E-4 2.0E-3 1.6E-2	5.4E-2 1.2E-1 5.4E-1
	One carbon pool by folate DNA replication	KEGG-pathway	3.4E-2 6.1E-2	5.7E-1 5.3E-1
Pink	melanosome membrane autophagosome membrane	GOTERM	1.6E-2 3.8E-2	6.8E-1 7.4E-1

^*^P-value: modified Fisher Exact *p*-value, ^**^Benjamini: Benjamini-Hochberg false discovery rate (FDR) adjusted *p*-value.

## DISCUSSION

Although single-target drugs inhibit or activate a specific target, their effects may be sub-optimal because of compensatory mechanisms [14–17]. Therefore, multi-target dugs are preferred to deal with the complexity of diseases [14, 16–18]. Cancer cell types are commonly classified by histopathology as well as molecular characteristics like gene expression, mutations, copy number variations and epigenetic alterations. These molecular characteristics help identify cancer-type and stage-specific prognostic biomarkers. In comparison to cancer type-specific biomarkers, multi-cancer biomarkers are more precise and accurate in research and clinic [11].

Previously, various specific biomarkers have been described for HNC or CC [19, 20]. However, consensus biomarkers are not well known for HNC and CC. Therefore, we investigated the various consensus gene modules in HNC and CC. We identified 8 consensus gene modules that showed differential expression patterns between cancer and normal samples in both types of cancers. Each module contained common genes that were important in HNC and CC. For example, SMAD2 that was included in the brown module correlated with poor prognosis in oral SCC [21] as well as cell cycle regulation and epithelial to mesenchymal transition (EMT) in CC [22]. Moreover, well known molecular biomarkers of HNC such as IL8, MMP1, and MMP10 [20] were included in the blue and green modules (Supplementary Table 1). Some of the genes in the modules are well known in various cancers, but have not been fully investigated in HNC or CC. For example, CACYBP in the brown module correlates with proliferation and metastasis in colon cancer [23, 24] as well as drug resistance in pancreatic cancer [25]. The modules also contained genes like LRRN4CL, NAV3 and STMN1 that have not yet been investigated in cancer research. Functional studies of these genes, which are not well known in HNC and CC will potentially reveal novel molecular mechanisms for both cancers and identify new molecular targets for the diagnosis and treatment.

We also explored the biological functions of each module by GO terms and KEGG pathway annotation. GO terms such as cancer initiation and progression, chemotherapy, cell cycle, immune response, tetrahydrofolate metabolic process and cell adhesion molecules were included in the red and black modules. Functional enrichment analysis identified cancer cell migration, invasion and survival as common pathways.

In the brown module, ATP binding was a significant term with many ATP binding-associated genes like NIMA related kinase 2 (NEK2). NEK2 regulates centrosome separation by phosphorylating centrosomal proteins [26–28]. Aberrant NEK2 activity has been investigated in various malignancies [29–31] including CC [32, 33] and HNC [34]. In the turquoise module, extracellular exosome was identified as a significant term. Extracellular exosome is an organelle that contributes to intercellular communication and is produced by all cell types [35, 36]. It is implicated in the progression of various cancers, including brain and head and neck cancer [37–40]. The turquoise module included 33 extracellular exosome associated genes. These included LYN, a member of the SRC family of protein tyrosine kinases. Lyn is a key mediator of cell proliferation, migration and invasion in CC [41] and HNC [42].

The type I interferon (IFN) signaling pathway, which is involved in the antiviral response [43], host immunity [44] and cytotoxicity [45] was a significant term of the green module. IFNs belong to a family of multifunctional cytokines that activate (Janus Kinases)/STAT (Signal Transducer and Activator of Transcription) signaling pathway [46, 47], which up-regulates IFN-stimulated genes (ISG) [48]. ISG15 was localized in the green module [49].

A limitation of this study is that we used genes with significantly different expression patterns between cancer and normal samples for identifying consensus module. In future studies, we plan to pursue the whole gene set to identify the consensus modules that will also include similarly expressed genes with aberrant function due to mutations. Future studies will also include the validation of the identified gene modules using other cancer types.

In conclusion, we identified consensus gene modules of HNC and CC to identify common targets for multicancer therapy, especially for cancers that are HPV16-positive. The modules included genes that are involved in significant biological functions associated with cancer progression.

## MATERIALS AND METHODS

### Gene expression dataset analysis

We used a publicly available gene expression dataset (GSE6791) [50] that included 42 head and neck cancer, 14 normal head and neck, 20 cervical cancer and 8 normal cervix tissue samples. The HPV types in these cancer samples were HPV16, HPV18, HPV33, HPV31, HPV35, HPV55 and HPV66. We analyzed HPV16-positive samples only to exclude bias due to different HPV types. The dataset is summarized in Table 3.

**Table 3:** Summary of the dataset used in this work

Dataset	Platform	Number of samples and probes	Number of HPV16 positivesamples
GSE6791 [14]	HG-U133Plus2	42 HNC, 14 normal tissues of head and neck,20 CC, 8 normal tissues of cervix, 54675 probes	13 HNC, 8 CC

^*^HNC: Head and neck cancer; CC: Cervical cancer; HPV: Human papilloma virus.

### Statistical analysis

Mann–Whitney U test was performed to determine the differently expressed genes in HNC and CC cancer samples in relation to their corresponding controls. After identifying the differentially expressed genes in HNC and CC, hierarchical clustering analysis was performed to construct different modules, as described previously [7, 12]. Principal component analysis (PCA) was used to identify the eigengene of each cluster or module. All statistical analyses were conducted using Rversion 3.3.1 software package including packages for consensus module detection.

### Consensus module construction

Gene modules refer to genes that show similar expression patterns in cancer cells or tissues in comparison to normal cells or tissues. Consensus modules refer to modules that are similar in multiple cancers. Hierarchical clustering according to a measure of dissimilarity is used to group genes with similar expression profiles into modules [12]. We used average linkage hierarchical clustering with consensus dissimilarity measure and defined modules as branches of a tree [7, 51]. For cut-off branches, we used a fixed height cut-off value of 0.95. The modules contained a minimum number of genes (25 genes per module in this study).We identified modules in a multistep process [7]. First, we performed hierarchical clustering based on consensus dissimilarity measure. Then, the cluster tree was cut at a fixed height cut-off value. Each branch was considered a separate module. Genes that were not assigned to any branch or module were denoted in grey.

### Construction of the eigengenes network

We performed principal component analysis (PCA) to identify eigengenes in the consensus gene modules. PCA is a nonparametric statistical method that reduces data dimensionality and converts correlated variables into uncorrelated variables called principal components [52, 53]. We calculated principal components of each gene module. The first principal component is called an eigengene and represents the module. Each principal component is represented in the form of linear combinations of gene expressions in the module according to the following formula:

eigengene=C1g1+C2g2+C3g3+·····+Cngn

where g_1_, g_2_, g_3_… g_n_ are gene expressions, and C_1_, C_2_, C_3_…C_n_ are weights of each gene expression.

### Module preservation and biological validation of modules

Module preservation statistics were used to evaluate if a module defined in one data set was also present in another data set. The preservation among modules was evaluated by the correlation of eigengenes of each module [51]. The preservation of eigengenes between the *i*^th^ and *j*^th^consensus modules in data sets A and B were calculated as

$$preserv_{ij}^{\left(A,B\right)}=1-\frac{\left|cor\left(E_{i}^{\left(A\right)},E_{j}^{\left(A\right)}\right)-cor\left(E_{i}^{\left(B\right)},\;E_{j}^{\left(B\right)}\right)\right|}{2}$$

where $E_{i}^{\left(A\right)}$ and$E_{i}^{\left(B\right)}$denote the eigengenes of the *i*^th^consensus module in data sets A and B, respectively; *cor(X,Y)* represents correlation coefficient of X and Y.

High values indicate strong preservation between the *i*^th^and *j*^th^consensus modules across the two data sets. The preservation statistic is maximized when the correlation of the *i*^th^and *j*^th^consensus modules in data set A is the same as in data set B. For biological validation, we used the KEGG pathways to determine the consensus biological terms that were associated with the gene lists in modules [54].

## SUPPLEMENTARY MATERIALS TABLE





## Abbreviations

SCC: Squamous cell carcinoma
HPV16: Human papillomavirus type 16
PCA: principal component analysis
HNC: head neck cancer
CC: cervical cancer
GO: gene ontology
FDR: false discovery rate
NEK2: NIMA related kinase 2
IFN: interferon
STAT: Signal Transducer and Activator of Transcription
ISG: IFN-stimulated genes
