# Supplementary material for: Highly preserved consensus gene modules in human papilloma virus 16 positive cervical cancer and head and neck cancers
Source: Oncotarget. 2017 Dec 7;8(69):114031–40. doi: 10.18632/oncotarget.23116 (PMC5768383; doi:10.18632/oncotarget.23116)
Supplement: Supplementary file 1 [file oncotarget-08-114031-s001.pdf]

Highly preserved consensus gene modules in human papilloma virus 16 positive cervical cancer and head and neck cancers

SUPPLEMENTARY MATERIALS

Supplementary Table 1: Summarizes the entire gene lists of 8 consensus models. See_Supplementary_Table 1.
